# The prevalence of complementary and alternative medicine use in the general population of Babol, North of Iran, 2018

**DOI:** 10.1186/s12906-021-03281-7

**Published:** 2021-04-08

**Authors:** Reihaneh Moeini, Seyyed Ali Mozaffarpur, Morteza Mojahedi, Seyed Davoud Nasrolahpour Shirvani, Narjes Gorji, Roshanak Saghebi, Farid Abolhassani Shahreza, Hoda Shirafkan

**Affiliations:** 1 Traditional Medicine and History of Medical Sciences Research Center, Health Research Institute, Babol University of Medical Sciences, Babol, Iran; 2 Healthcare Services Management, Department of General Courses, School of Medicine, Social Determinants of Health Research Center, Research Institute for Health, Babol University of Medical Sciences, Babol, Iran; 3 Department of Traditional Medicine, School of Traditional Medicine, Tehran University of Medical Sciences, Tehran, Iran; 4 Internal Medicine, National Institute for Health Research, Tehran University of Medical Sciences, Tehran, Iran; 5 Social Determinants of Health Research Center, Health Research Institute, Babol University of Medical Sciences, Babol, Iran

**Keywords:** Persian medicine, Complementary therapies, I-CAM-Q

## Abstract

**Background:**

Complementary and Alternative Medicine (CAM) have recently become more popular and accepted worldwide. One principal step to identify the status and organize strategies of CAM is evaluating the manner and the prevalence of its usage among people. This study aimed to investigate the prevalence of CAM modalities usage by the people of Babol, a central city in the North of Iran, in 2018.

**Methods:**

Using the original International CAM Questionnaire (I-CAM-Q), a questionnaire was redesigned in Persian (Farsi) with some changes such as adding special modalities in Iran and its validity and reliability were assessed. Six hundred households were evaluated using a cluster sampling method in 2018 spring by 12 trained interviewers.

**Results:**

Finally, 1770 questionnaires were correctly completed. A total of 110 participants (6.21% of the completed questionnaires) had visited CAM therapists in the last year, 109 persons (6.15%) had received prescriptions from physicians and paramedics to use CAM, and a total of 1032 people (58.30%) used herbs and herbal medicines in the last 12 months. Also, 1265 individuals (71.46%) had used CAM throughout their lives. The most popular methods were herbal medicine (65.76%), Persian Medicine (13.78%), water therapy (10.45%) and music therapy (8.36%). The use of CAM was more popular among women.

**Conclusions:**

The general use of CAM in Babol was similar to other studies, but there were fewer visits by CAM therapists and less frequent adoption of common methods including homeopathy, acupuncture, and energy therapy. It was found that CAM was mostly used for non-serious diseases such as cold and transient gastrointestinal disorders, a pattern that is different from other studies in this field.

**Supplementary Information:**

The online version contains supplementary material available at 10.1186/s12906-021-03281-7.

## Background

Complementary and Alternative Medicine (CAM) is commonly considered as therapeutic approaches which is not part of conventional medicine [1]. Herbal medicine, traditional medicine, homeopathy, chiropractic, acupuncture, reflexology, and massage are among the most well-known CAM modalities all over the world. CAM has become more popular and accepted in recent decades in both developed and developing countries [2]. In such instances, 74% of Canadians and 60% of Chinese have had the experience of using CAM methods [3]. The prevalence of CAM is not directly comparable across countries and surveys due to various definitions and instruments used. Also, cultural and socioeconomic factors determine various trends in CAM use; as an example, the use of CAM in 1 year in Europe was reported from 10% in Hungary to almost 40% in Germany; but, an increasing trend in CAM usage seems to be obvious [4–6]. However, the tendency to use CAM did not grow in all modalities equally [7]. Indeed, cultural and social differences can also affect the choice of the method used [8]. These kinds of medicine are used for various purposes such as therapy, disease prevention and maintenance of well-being [2].

Many studies have been conducted across countries, cities, and continents to determine how people are using these methods and investigate their attitudes towards them [9]. These are the first step in identifying the situation and planning the strategies to promote the safe usage of CAM, as well as preventing or reducing undesirable consequences of these methods. Indeed, the principal step to identify the status and organize strategies of CAM is evaluating the manner and frequency of its usage among people [3].

In Iran, due to the existence of particular traditional medicine [Persian Medicine; PM] with a history of several thousand years, people are still using these methods [10]. Moreover, since 2007, a specialized field of Persian medicine was established as a PhD program in some medical universities and interested physicians entered this field. Despite that, many non-academical practitioners are active in this field l as medicinal herbs sellers (Attari) or traditional healers such as bone setters who correct joint dislocations and treat musculoskeletal problems. In addition, from 2016, some preventive recommendations of PM entered in the first level of health services in 10 cities of Iran (Health houses) as a pilot study. There are few official therapists in other common methods of CAM in the world such as homeopathy, chiropractic and acupuncture in Iran [11]. Although some studies have been conducted on the use of CAM methods among different patient groups in Iran, studies on the general population are limited, and most of them have used researcher-made questionnaires. Examples include the study by Lotfi et al. in 2014 that examined the extent of the use of different types of CAM by Kashan population and their attitude toward them [3].

Babol city is the oldest city in northern Iran with a history of 3000 years, which had been the capital of Mazandaran province for many years. The first university of medical sciences in northern Iran was established there. Also, currently it has the only university with a faculty of Persian medicine in northern Iran. Furthermore, it was among 10 cities applying PM preventive recommendations in health houses. Babol county includes the city of Babol and several surrounding villages, and it is the most populous county in northern Iran. The population is almost equally divided into city (250000) and village (280000). Considering the lack of studies in this area and the impact of culture on the use of CAM, this study aimed to investigate the prevalence of the use of CAM and its different methods by the people of Babol county (Babol city and its villages(, in 2018, using a standard questionnaire. Moreover, exploration of the relationship between CAM use and user’s characteristics as well as data about sources of familiarity with these methods and out-of-pocket spending are other purposes of the study.

## Methods

### The format of the CAM Questionnaire (I-CAM-Q)

The International CAM Questionnaire (I-CAM-Q) is a unified tool that can provide comparable results for studies conducted worldwide to measure the use of CAM. I-CAM-Q was developed in an international group of CAM researchers as a standardized questionnaire to reduce questionnaire-related bias [12].

Using this questionnaire is common in several surveys on the utilization of CAM conducted in several countries [13–15]. As the type and circumstances of CAM may vary widely based on differences in the cultural backgrounds of each country, an adapted version of the I-CAM-Q is usually developed before implementation [16].

The sections of the Questionnaire together with the procedures of completion were thoroughly described in Quandt et al. study [12]. However, considering that in Iran special methods have been developed due to the prevalent use of PM, using the original I-CAM-Q for a survey of CAM use without adapting it to the Iranian culture did not seem reasonable to the researchers. In addition, according to previous studies, some of the commonly used CAM therapies in Europe or East Asia are not applicable in Iran [17, 18]. Therefore, it had to be localized; to this end, in the first step, the English questionnaire was translated into Persian and during three sessions with the presence of 5 traditional medical specialists and one health specialist, common Iranian methods were included and uncommon methods were excluded. In Table 1 of the questionnaire, herbalist and spiritual healer were removed and traditional medicine healer, bone setter and massage practitioner were added. In Table 2, manipulation and homeopathy were removed and natural supplement, wet cupping, leech therapy, bloodletting, dry cupping and water therapy, and music therapy were added. In Table 3, homeopathic remedies were removed and oral natural supplement and non-oral natural supplement were added. In Table 4, meditation and relaxation were merged, qigong and visualization were removed and cupping, music-therapy, massage, water therapy, detox pad and PM were added. Also, since the four sections of the original questionnaire considered the use of CAM only over the past year, another section was added with the same pattern for investigating the use of different types of CAM throughout life.

**Table 1:** Basic characteristics of participants

	Characteristics	frequency	percent
Age (year)	0–10	211	11.92
	11–20	273	15.42
	21–30	264	14.91
	31–40	303	17.11
	41–50	282	15.93
	51–60	223	12.59
	61–100	200	11.29
	Missing data	14	0.79
Sex	Female	891	50.33
	Male	879	49.66
Education	Under 7 years old	135	7.62
	Illiterate or Elementary school	602	34.01
	Middle School	305	17.23
	High school	436	24.63
	Associate Degree or bachelor’s degree	242	13.67
	Master’s degree and higher	40	2.25
	Missing data	10	0.54
Occupation	Official employee	93	5.36
	Official worker	27	1.46
	Housewife	559	31.58
	Self-employment	318	17.90
	Farmer and gardener	85	4.74
	Retired	57	3.16
	Under 7 age-old	135	7.62
	Student	347	19.54
	Workless	27	1.18
	Other	99	5.31
	Missing data	13	0.73
Marriage status	Single	618	34.91
	Married	1066	60.22
	Divorced	14	0.79
	Widow and widower	47	2.65
	Missing data	35	1.97
responder	Him/Her self	1244	70.28
	Parents	453	25.59
	Child	46	2.59
	Supervisor	21	1.18
	Missing data	6	0.33

**Table 2:** Visit by Healthcare providers in the last year

health care providers	Visited^a^ (%)	Motivation^b^ (%)^c^			Helpfulness (%)^c^			
		Acute illness	Long-term illness	Improvement of well-being	Very	somewhat	Not at all	Don’t know
Family practitioner	664 (37.51)	466 (70.18)	158 (23.79)	34 (5.12)	313 (47.13)	303 (45.63)	45 (6.77)	3 (0.45)
General Physician	518 (29.26)	381 (73.55)	124 23.93	19 (3.66)	218 (42.08)	285 (55.01)	15 (2.89)	0 (0.00)
Specialist Physician	670 (37.85)	191 (28.50)	419 (62.53)	14 (2.08)	302 (45.07)	330 (49.25)	11 (1.64)	2 (0.29)
Nutritionist	19 (1.07)	6	9	7	11	5	3	0
Traditional medicine healer	67 (3.78)	19 (28.35)	33 (49.25)	17 (25.37)	21 (31.34)	30 (44.77)	13 (19.40)	3 (4.4)
Acupuncturist	9 (0.50)	2	7	0	5	3	1	0
Bone setter	14 (0.79)	13	1	0	11	3	0	0
Massage practitioner	14 (0.79)	1	9	4	10	3	1	0
Other	9 (0.50)	3	4	2	3	3	2	1

^a^Percentages are calculated relative to the total sample population (Column percentage)

^b^Percentages are calculated according to the type of health care provider (Row Percentage)

^c^Percentages have been reported only for cases that have used more than 20

**Table 3:** CAM treatments received by practice or advice of physicians in the last year

CAM treatments	Received	common reasons for CAM treatments	Helpfulness (%)^a^			
			Very	Somewhat	Not at all	Don’t know
Medicinal herbs and herbal medicine	61	Cold and respiratory problems, Musculoskeletal problems, diabetes	38(62.29)	19(31.14)	2(3.27)	2(3.27)
Natural supplement	11	Cold, anemia	10	1	0	0
Wet cupping	21	Cough, Musculoskeletal problems, skin problems	6(28.57)	11(52.38)	4(19.04)	0(0.00)
Leech therapy	2	Musculoskeletal problems, skin problems	1	1	0	0
Bloodletting	1	Blood concentration	1	0	0	0
Acupuncture	4	Weight modification	2	2	0	0
Yoga	1	Relaxation	1	0	0	0
Massage	5	Musculoskeletal problems	3	2	0	0
Dry cupping	5	Musculoskeletal problems	3	2	0	0
Water therapy	19	Musculoskeletal problems	10	7	2	0
Other	5	–	3	2	0	0
Total	135	–	78(57.77)	47(34.81)	8(5.92)	2(1.48)

^a^Percentages have been reported only for cases that have used more than 20.

**Table 4:** Use of dietary supplements in the last year

Types of dietary supplements	Number	%	Common reasons	Helpfulness			
				Very	somewhat	Not at all	Don’t know
Oral usage of medicinal herbs and herbal medicines	1032	58.30	flu and cold, digestive tract problems, prevention and improvement of health, nervous system and psychiatric problems, to change body temperament	64.92	30.03	1.9	3.15
Vitamins and minerals	335	18.9	Musculoskeletal problems, anemia and iron deficiency, general strengthening and well-being	35.85	50.38	5.52	8.25
Oral usage of Natural products	365	20.6	colds and upper respiratory tract infections, general strengthening and well-being	67.34	13.68	8.33	10.65
Non-oral usage of Natural products	374	21.7	prevention and maintenance of health, colds and respiratory tract infections, musculoskeletal and skin disorders	56.20	37.59	1.27	4.94

### Assessing validity and reliability

In order to assess the face validity, a pilot study was carried out with 20 individuals at Babol University of Medical Sciences between Jun. 2018 and Feb. 2018. To prevent any misunderstanding, some terms were revised. After this step, the questionnaire was revised by 10 CAM specialists to confirm the validity. The reliability of the questionnaire was tested by a test-retest method with two stages of completion of the form by 30 participants in a two-week interval via Cronbach’s alpha.

In the last part of the questionnaire, a few questions about attitude to CAM, the amount of spending on CAM and familiarity with the methods were added. In this part, if the participants did not use complementary therapies at all, they were asked to choose from three important reasons from available answers. If any of the methods of CAM were used, participants were asked about the reasons for using it, the cost and familiarity with the method.

### Sample size calculation

By the Cochran’s formula for sample size calculation in prevalence studies, a minimum sample size of 1537 individuals was calculated. A prevalence of 80% of CAM use among people according to previous studies in Iran [14], type I error 5% and precision of 2% was assumed. Considering a dropping rate of 15% for incomplete or incorrect questionnaires, the sample size became 1768 individuals. By considering an average family size of three, approximately 600 households were included in this study.

### Study population and procedures

This large population-based study was conducted between April and May 2018 in Babol, Mazandaran, Iran. The participants were all community residents who had been living in Babol, were able to participate in the study, and singed written informed consent. For children under the age of 18 and disabled persons who could not be interviewed, their parents or caregiver sign the written informed consent and also replied the questions. The inhabitants living in students’ dormitories, military posts, and nursing homes were excluded.

Based on the recommendations of the World Health Organization and the Demographic and Health Surveys (DHS) Country Planning Implementation Model, this sample size was divided into 60 clusters. The headings of 60 clusters were randomly selected using the latest household census data available at the Babol Health Center covering urban, rural and mobile populations [19]. All of those who were at home at the time of the visit were included in the study. The face-to-face interviews at the participants’ homes were conducted by thoroughly trained interviewers.

The purpose of this study was to investigate the use of CAM in all ages, different levels of literacy and urban and rural areas; thus, contrary to the standard questionnaire which was completed in a self-report manner, our questionnaire was completed by trained interviewers via face-to-face interviews. Therefore, twelve interviewers with experience in health censuses were selected by an expert from the health network, and they were trained for 9 h in three sessions, and then each of them was asked to complete the questionnaires for five cases as the pilot stage and submitted it to the analyst.

### Statistical analyses

The collected data were analyzed by SPSS (version 25). The participants’ socio-demographic characteristics include age, gender, marital status, education level, occupation, and insurance type. For descriptive analysis, frequencies (percentage) and means (± standard deviation) were calculated. To compare the quantitative explanatory variables in the two groups, we ran t-test and nonparametric test of Mann-Whitney U, and for qualitative variables, we performed chi-square and Fisher exact tests. In order to evaluate the relationship between CAM use and other covariates (including gender, age, education, residency location, and marital status), binary logistic regression was used. Using CAM in the past 12 months was the dependent variable. The backward elimination technique was used to fit the regression model. The adjusted odds ratios (OR) and its 95% confidence intervals were calculated. The significance level was set to 0.05.

### Ethics and consent

The research was approved by the research ethics committee of National Institute of Health Research (reference number: IR.TUMS.NIHR.REC.1396.53). The written informed consent was signed by every participant. For disabled persons and children under the age of 15, their legal guardians or parents signed the informed consent.

## Results

Six teams completed interviews in 23 days in May and April 2018. With reference to 600 households, the initial population was estimated to be 1960, of which 75 were absent despite repeated visits and 72 were reluctant to cooperate. From the 1813 collected questionnaires, 1770 questionnaires were correctly completed. Totally, 849 participants (47.96%) lived in the urban areas and 913 (51.58%) in the rural areas. The average age of participants was 35.53 ± 20.26. Eight hundred seventy-nine (49.66%) of participants were males and 891 (50.33%) were females. Two hundred and eighty-five (16.10%) of them had received a university education (Table 1). According to available population information from the county, the study population was representative to the general population in terms of age, sex and residency, but there is a difference only in terms of education. Indeed, the ratio of people with university education in our study was less than this ratio in the population of the city.

### Healthcare provider

The total number of persons who had visited conventional therapists and CAM therapists during the last year was 1347 (76.10%) and 110 (6.21%) respectively. Visiting conventional therapists was significantly more frequent in women (*P* = 0.037). Also, in rural areas, CAM therapists are more commonly referred (*P* = 0.003). The most common cause for visiting by CAM therapists was complications of bone and joint pain (44 visits). Furthermore, among the CAM therapists, visiting traditional therapists was the most frequent. Also, the most common causes of visiting traditional therapists were bone and joint pain, colds and respiratory problems, skin and hair problems, and blood concentrations, respectively. The highest degree of satisfaction with complementary therapists was associated with the two groups of bonesetters (78.57%) and massagers (71.42%) (Table 2).

### CAM treatments received from physicians

One hundred and thirty-five prescriptions have been made for 109 persons for the use of CAM therapies by conventional physicians and Paramedics. The most frequent prescriptions were for the use of medicinal herbs and herbal medicines in 61 cases (3.46% of all participants), wet cupping in 21 cases (1.18%) and water therapy in 19 cases (1.07%).

Twenty--eight prescriptions (20.7% of all prescriptions) were made by a physician specialized in traditional medicine. However, 46 (34.47%) cases were performed by specialists in other sub-disciplines of medicine, 2 (1.48%) by nutritionists and 59 cases by general practitioners and family physicians. (Table 3).

### Use of dietary supplements

A total of 1032 people (58.30%) used herbs and herbal medicines orally in the last 12 months with the aim of treating illness or improving health. Over the past 3 months, 952 (53.78%) have used this method from which 225 people (12.71%) have used medicinal plants daily: 63.46% for the flu and cold, 30.71% for the digestive tract problems, 32.7% for prevention and improvement of health, 12.40% for the nervous system and psychiatric problems, 7.46% to make body temperament (Mizaj) cold and 3.77% to make body temperament warm.

Regarding the herbs, the respondents have used *Mentha piperita* (20.4%), thyme (*Thymus vulgaris*) (11.4%), *Mentha pulegium* (11.4%), *Citrus aurantium* flower (10.7%), *Cinnamomum zeylanicum* (9.8%), *Mentha aquatic* (9.4%), and *Echium amoenum* (8.7%). Most of the mentioned plants are grown as wild herbs or cultivated in this area for its favorable climate.

A total of 335 (18.9%) respondents used a variety of vitamins and minerals. Iron supplements, Ca supplements and folic acid were the most commonly used supplements.

In order to treat, improve, or/and maintain health, 20.6% of the participants have used natural products in the past year. Three hundred and thirty participants (18.6%) have used these products in the last 3 months and 74 (4.18%) people used these products on a daily basis.

The typical natural product is honey, which is used 199 times alone or in combination with other products. Of these, 65 cases were lemons and honey, which were used to prevent or treat colds and upper respiratory tract infections. Other consumed natural products to improve health were rock candy and hot water (*n* = 100, 5.64% of participants), grape juice (*n* = 37, 2.09%) date sauce (*n* = 15, 0.84%), red sugar (*n* = 19, 1.07%), royal jelly (*n* = 7, 0.39%), persimmon juice (*n* = 11, 0.62%), medlar juice (*n* = 9, 0.50%) and sesame (*n* = 7, 0.39%).

Three hundred and seventy-four people (21.7% of the total) used medicinal herbs and natural products non-orally, such as ointment, fumigation or smoke. Among these medical herbs, *Heracleum persicum* smoke (12.9%, *n* = 230), *Cucurbita pepo* (pumpkin) fumigation (3.4%, *n* = 77), and *Eucalyptus* fumigation (0.8%, *n* = 15) have been used.

Totally, 1177 people (66.6%) used one of the herbal products and natural food supplements (except minerals and vitamins), either orally or topically in the last 12 months (Table 4).

### Self-help practices

Only a total of 28 (1.58%) persons have used one of the methods listed in Table 3 of original I-CAM questionnaires including yoga (3 persons), relaxation and meditation (2 persons), and prayer therapy (24 persons). Because it was predictable due to cultural differences, other methods had been added, such as music therapy and massage or dry cupping at home by one’s own or other relatives. This table also required information on the observance of special dietary guidelines of PM which showed a notable percentage (10.39%, *n* = 184) compared to other methods. However, Vow and charity and participating in religious ceremonies obtained the most stats (44.57%). The highest percentage of good satisfaction (very helpful) was related to music therapy (78.23%, *n* = 115), dietary advice (70.1%, *n* = 129) and massage (67.07%, *n* = 55). (Table 5).

**Table 5:** Self-help practices used in last year

CAM treatments	Used (%)	Reasons
Cupping	30 (1.69)	Musculoskeletal problems
Massage	82 (4.63)	Musculoskeletal problems
Dietary recommendations of persian medicine	184 (10.39)	Gastrointestinal problems
Music	147 (8.30)	Relaxation
Relaxation and Meditation	2 (0.1)	Relaxation
Yoga	3 (0.15)	Relaxation
Ti- chi	0 (0.00)	–
Water therapy	78 (4.40)	Musculoskeletal problems
Praying	19 (1.07)	Disease prevention
Vow, charity and participating in Religious ceremonies	789 (44.57)	Disease prevention
Detox pad	12 (0.67)	Musculoskeletal problems

There was a significant relationship between education level and using music therapy (*P* < 0.001) and dietary recommendations (*p* = 0.05). There was no significant relationship between gender and living area with using any of these methods (*p* > 0.05).

Using logistic regression showed that education, age and sex had a significant relationship with using music therapy.

Other ORs and their 95% CI were reported in Additional file 1: Appendix 1.

### Use of complementary medicine throughout life

**Fig. 1:**
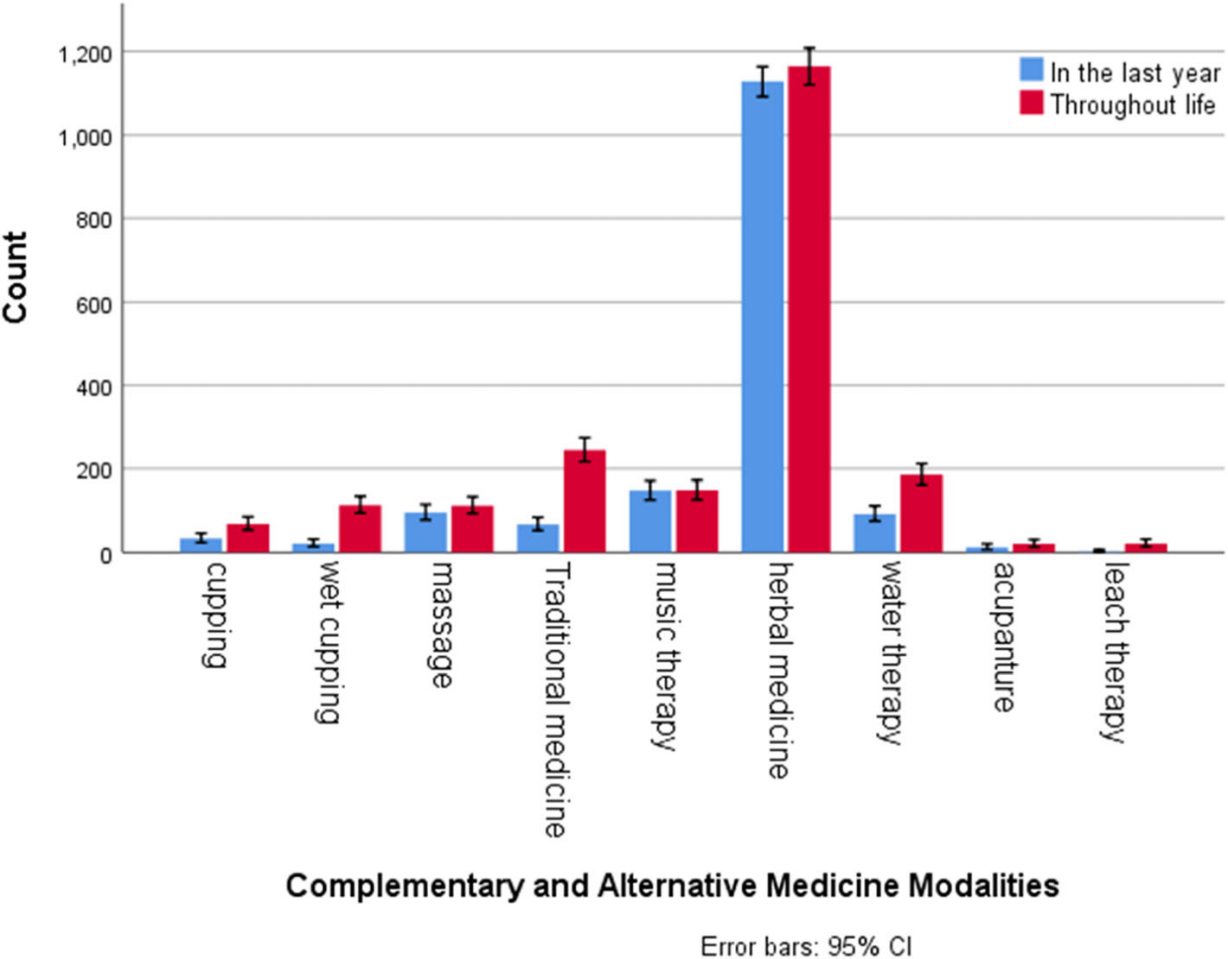
Use of Complementary And Alternative Medicine in the last year and throughout life

As mentioned above, one table was added to the standard questionnaire to examine the extent of using CAM throughout life. More detail was presented in Table 6 and Fig. 1.

**Table 6:** Use of complementary medicine throughout life

CAM Methods	Frequency (percent)	Common reasons	Helpfulness (%)^a^			
			Very	Somewhat	Not at all	Not determined
Persian medicine	244 (13.78)	Gastrointestinal disorders, Cold and respiratory system disorders, health improvement	158 (64.75)	79 (32.37)	2 (0.81)	5 (2.04)
Herbal medicine	1164 (65.76)	Cold and respiratory system disorders, gastrointestinal disorders, health improvement	711 (61.08)	403 (34.62)	21 (1.80)	29 (2.49)
Wet cupping	113 (6.38)	Blood concentration, health improvement, musculoskeletal disorders	66 (58.40)	32 (28.31)	10 (8.84)	5 (4.42)
Dry cupping	68 (3.84)	musculoskeletal disorders, health improvement	41 (60.29)	21 (30.88)	6 (8.82)	0 (0.00)
Bloodletting	4 (0.22)	Blood concentration	2	1	1	0
Leech-therapy	21 (1.18)	musculoskeletal disorders, Blood concentration	7 (33.33)	9 (42.85.)	1 (4.76)	3 (14.28)
Acupuncture	20 (1.12)	musculoskeletal disorders, body weight correction	6 (30.00)	7 (35.00)	6 (30.00)	1 (5.00)
Yoga	8 (0.45)	Relaxation and improvement of mental status	3	4	1	0
Massage	112 (6.32)	musculoskeletal disorders	72 (64.28)	35 (31.25)	5 (4.46)	0 (0.00)
Bonesetter	21 (1.18)	musculoskeletal disorders	13 (61.90)	6 (28.57)	2 (9.52)	0 (0.00)
Energy Therapy	4 (0.22)	Relaxation and improvement of mental status	1	2	1	0
meditation	2 (0.11)	Relaxation and improvement of mental status	1	1	0	0
Music therapy	148 (8.36)	Relaxation and improvement of mental status	126 (85.13)	19 (12.83)	0 (0.00)	3 (2.02)
Hypnotism	2 (0.11)	Relaxation and improvement of mental status	1	1	0	0
Water therapy	185 (10.45)	musculoskeletal disorders, health improvement	126 (68.10)	47 (25.40)	1 (3.70)	0 (0.00)
praying	37 (2.09)	health improvement	17 (45.94)	15 (40.54)	0 (0.00)	5 (13.51)
Aromatherapy	3 (0.16)	Relaxation and improvement of mental status	2	1	0	0
Homeopathy	0 (0.00)	–	0	0	0	0
Ayurveda	0 (0.00)	–	0	0	0	0
Total CAM	1265 (71.46)					

^a^Percentages have been reported only for cases that have used more than 20.

There was no significant relationship between age groups, level of education, occupational status, living area and marital status and insurance with life-long CAM usage, but a significant relationship between the use of CAM therapies among two sexes was detected (p = 0.03) in which 63.93% (*n* = 562) of men and 79.12% (*n* = 705) of women have used CAM in their lives. Using logistic regression showed that gender and marital status had a significant relationship with using CAM modalities throughout life. Age, education and residency location were removed from the regression model in steps 1,2 and 3, and it was revealed that men were half as likely to use CAM (OR = 0.55, *p* < 0.001, CI: (0.45, 0.67)) when compared to women. Also, married people used CAM, about 30% more than the singles (OR = 1.32, *p* = 0.008, CI: (1.08, 1.63)).

Fifty-eight percent (*n* = 510) of men and 73.3% (*n* = 654) of women had used herbal medicine. This difference was significant as well (*p* = 0.004). Using logistic regression showed that gender had also a significant relationship with using herbal medicine throughout life. Age, education, residency location and marital status were removed from the regression model in steps 2, 3, 4 and 5 and the result revealed that men are half as likely to use CAM (OR = 0.51, *p* < 0.001, CI: (0.41, 0.62)) when compared to women.

One hundred and thirteen (6.4% of all and 8% of people over the age of 20) had used wet cupping in which 68.14% (*n* = 77) of wet cupping users were males and 31.85% (*n* = 36) were females. In fact, 8.8% of all men and 4.2% of women had used this method, which is statistically significant (*p* = 0.01). Seven percent of those who had a history of wet cupping was less than 15 years of age.

In our study, a significant difference was seen (*p* = 0.02) between using music-therapy among urban residents (10.12%, *n* = 86) and rural residents (6.79%, *n* = 62). Also, the level of education significantly influenced the use of music therapy (*p* < 0.001). Furthermore, women (1.79%, n = 16) used acupuncture more than men (0.45%, *n* = 4) (*p* = 0.008).

Also, 26.5% (*n* = 336) of users of CAM are under the age of 20 which was mostly due to the use of herbs (88.69%). Moreover, 6.19% (*n* = 30), 3.30% (*n* = 16) and 1.23% (*n* = 6) of those less than 20 years had used water therapy, massage and wet cupping respectively.

Using logistic regression showed that dry cupping had a significant relationship with age, and gender, wet cupping with gender, marital status and education, massage with marital status and education, Persian medicine with location, gender and education, music therapy with location, age and education, water therapy with location, education and marital status and acupuncture with age and gender. The ORs were reported in Additional file 1: Appendix 2.

### Reasons for using or not using

Lack of enough information about CAM methods (47.5%, *n* = 264), no disease and no need for treatment (32.4%, *n* = 180), and lack of belief in these methods (29.5%, *n* = 164) were the main reasons for not using CAM in people who had never used these methods before.

The most common reasons for using complementary medicine were less complication from these methods (52%, *n* = 637), being more effective than the common treatments (43.2%, *n* = 525), the usefulness of these methods, the use of conventional medicine (32.4%, *n* = 394), and not having serious problem for referring to conventional medicine (19.6%, *n* = 239).

### Other findings

In our study, we found that 50.04% (*n* = 583) of users of medicinal herbs and natural products have satisfied their needs by themselves or their relatives. Also, 41.88% (*n* = 488) had bought them from herb seller (attari) and 3.43% (*n* = 40) from organic product stores.

Seventy-five percent (*N* = 950) of CAM users have paid less than 500.000 Rials for CAM including herbs and natural products. The average cost was about 400,000 Rials.

Sixty-three percent (*N* = 799) of CAM users selected family and relatives, 37.07% (*n* = 469) friends and 8.85% (*n* = 112) listening to radio and television as the method of acquaintance with CAM. Only 3.87% (*n* = 49) chose virtual space and 4.42% (*n* = 56) websites.

One hundred and fifty-nine participants (8.98%) reported receiving health advice based on PM from their local health workers over the past year.

## Discussion

This study is the first population-based Iranian study to have used an edited version of I-CAM questionnaire. The results of this study can be compared with similar studies in two ways: one is a comparison with other Iranian studies that did not use the International Standard Questionnaire and another in comparison with studies of other countries that used the International Questionnaire appropriate to their culture and circumstances.

Comparing the use of different methods of CAM throughout life and in the last year showed that such items as the use of herbal medicine and music therapy have been almost equal, indicating that these methods are part of the lifestyle of these people. However, in cases such as leech therapy, Persian medicine, wet cupping and acupuncture, which are a type of external intervention and need to see a therapist or service provider, the amount of use during a year will be much less than the use during a lifetime.

### Comparison with other Iranian studies

The results showed that the prevalence of using CAM during life was 71.46%. Prevalence of using medicinal herbs as a typical CAM was about 65.76% during life and 63.67% in the last year. Without considering medicinal herbs, the other methods of CAM was found to be 31.8% throughout life. Persian medicine, wet cupping, dry cupping, music therapy, and hydrotherapy were consecutively less popular.

One study in the capital of Iran (Tehran) in 2008 on 6148 individuals revealed that 66.3 and 52.5% of the study population have used at least one type of CAM throughout their lives and in the past year, respectively [20]. In another study in the west of Iran (Khorramabad) in 2013, total use of CAM and medicinal plants during lifetime was 79.9 and 69.2% respectively; furthermore, these were 58.2 and 37.7% in last year respectively [18]. The results of this study are more similar to the results of our study. However, it is lower for using medicinal plants and higher for energy therapy and prayer therapy. In another Iranian study in the northeast of Iran (Bojnourd) in 2015, 367 individuals were evaluated using clustered sampling. Totally, 84.65% of the subjects during their lifetime and 76.56% during the past year had used at least one CAM method [17]. In another study conducted in the center of Iran (Kashan) in 2014, from 576 individuals, 73.75% had a history of using one type of CAM methods during life. Medicinal plants use was the highest with 68%, and then there were cupping, massage and hydrotherapy [3]. The results of this study are nearly identical to ours. Generally, the results of the present study seem to be more similar to those of the studies conducted during the last 5 years. This may be due to the increased application and advertising of CAM in Iran. However, the use of chiropractic, homeopathy, hypnosis, and energy therapy in Babol is less than that in other cities that evaluated these methods. Lack of familiarity with these methods may be related to education level, as more than 50% of the study population of Bojnourd had a university education and only 17.5% were under diploma, but in Babol’s study only 16.1% of sample study (20.7% of those over 15 years old) had a university education. Also, the use of wet cupping in this study has been reported lower than some other studies in Iran [17, 18].

The reasons for using CAM methods such as wet and dry cupping as well as massage, in our study, have been similar to different studies, but the reasons for using other methods, such as herbs in the Babol study, are more similar to those in Bojnourd, where colds and gastrointestinal problems are at the forefront [17].

In a Subgroup analysis, there were significant differences between the use of medicinal herbs, acupuncture, and cupping by gender; the use of massage by marriage status; the use of music therapy by the living area and level of education; and the use of water therapy by marriage status and living area. Overall, the use of CAM, herbs, and acupuncture was higher in women and cupping in men. In Khorramabad study, most of the methods (except cupping) including medicinal herbs were significantly higher in women as well, and the use of acupuncture, yoga and energy therapy differed significantly between urban and rural communities. The use of acupuncture has also been more prevalent in college-educated people [18].

### Comparison with studies of other countries

The rate of the visit with one of the CAM therapists in this study was 6.2% which adds up to about 30% by adding medicinal herbs seller. Meanwhile, the visit with the conventional therapists was 76.6%.

A 2013 study by Albedah et al., using an edited version of I-CAM questionnaire in Kasim, Saudi Arabia, showed that 47.6% of participants had visited CAM providers in the last year (by eliminating prayer therapists). Visiting herbal medicine providers, honey vendors and wet cupping providers are the most frequent. The rate of visit with conventional medicine therapists was 83.4% [21]. Despite many cultural similarities, CAM services in the study of Albedah et al. still seems to be far more common, especially in reference to traditional therapists such as Hijamat (wet cupping). However, visiting with a homeopathic therapist in this study was approximately zero as well [21].

In one study in 2017 in Korea using the Korean version of I-CAM (completed by 1668 people online), visit with conventional physicians was 67.9% and with CAM providers were more than 20% [16]. Another study in 2011 in South Korea has reported that 69.3% of the respondents had visited TKM clinics one to four times during the previous year [22]. In a similar study using the German version of I-CAM completed in 2012 by 210 individuals, 44.3% of cases had visited with a CAM provider [23].

Overall, the frequency of visits with CAM therapists in the Babol study is far lower than in other studies, if not considering herbs sellers. Visiting the traditional medicine therapist in the Babol study is also far less common than in the studies of Korea and Germany [16, 23]. However, it should be borne in mind that those studies were conducted online and nationally. Notably, a comparison with Albedah et al. study conducted in one province of Saudi Arabia also confirms the lower rate of the visit with CAM providers in Babol.

As much as 6.1% of Babol study and 11.3% of Saudi’s study population had been prescribed to use CAM by the physicians and paramedics. The most frequent prescription was the use of herbs and herbal remedies (3.4 and 5.7% respectively) [18]. In the German study, 38.1, 7.2, and 14.9% of all participants were respectively prescribed by physicians to use herbal medication, acupuncture, and homeopathy [23]. In the Korean study, the rate of the most prescribed CAM method by physicians was 12.6% for supplements and 10.8% for acupuncture [16]. A comparison of the results showed that physicians’ recommendation for using CAM in Babol city is much lower than studies in Korea, Saudi Arabia, and Germany. This demonstrates the need to increase the familiarity of physicians of Babol with approved methods of CAM. However, no internal study was found to compare this data using this questionnaire.

In this study, only a total of 28 persons have used one of the methods listed in Table 3 of the original I-CAM questionnaires. Despite that, in the Saudi study, 10.7% used relaxation and 6.7% used meditation [22], and in the German study, more than 14% has used meditation, yoga, and Qigong methods [23]. Moreover, in the Korean study, yoga and meditation were the most frequent by 13.2 and 9.6% [13, 16]. As previously predicted, this information showed that many people in Babol are not familiar with these methods. Other methods such as special dietary guidelines of Persian medicine (10.4%) have been used instead.

In the Saudi study, 75% of respondents have used medicinal herbs for health and well-being over the past 12 months. In this study, about 25% of people used at least one type of vitamins and minerals including multivitamin, iron, folic acid, and B complex [21]. In the German study, 32.4% used homeopathic remedies, 40.2% used herbs and herbal remedies, and 41.2% used vitamins [23]. Overall, the use of vitamins and minerals in the Babol study sample appears to be less than the three other studies. On the other hand, the differences between the compounds used in the studies are interesting if the commonly used plants mentioned in the Saudi study are not included in the list of 40 plants in Babol.

Herbal medicine, homeopathy, chiropractic, and acupuncture were reported as the most frequently used CAM modalities in Europe [24]. The results of the Babol study comply with these results only in herbal medicine. Moreover, with regard to socio-demographics, CAM users are typically female, better-educated and middle-aged. These results are somewhat consistent with Babol study results. According to some global studies, musculoskeletal problems, depression, insomnia, severe headaches or migraines and gastrointestinal illnesses were the most typical conditions associated with CAM, whereas in the Babol study most types of the examined CAM such as massage, dry cupping and water therapy have been used to improve musculoskeletal problems. Overall, it seems that the people of Babol county are using some types of CAM, such as medicinal herbs, to a large extent, though mostly for simple diseases such as colds and transient gastrointestinal disorders. Yet, the use of many well-known CAM therapies in the world, such as homeopathy, chiropractic, energy therapy and acupuncture, is far below the global numbers. Self-care practices such as yoga and meditation are less commonly considered. Moreover, visiting CAM therapists was also extremely low in this area and it seems that people commonly use these methods as a self-treatment. Most of the plants used also come from the surrounding environment, which results in lower costs.

Our study had some limitations. Firstly, this is a cross-sectional study, so causality cannot be determined. Secondly, despite the frequent references to ask all participants, probably due to non-attendance or lack of cooperation of some, a suitable populated dispersion was not achieved in terms of education.

## Conclusion

This study, which was conducted for the first time in Babol, the most populated county of the north of Iran, showed that the use of medicinal herbs for the treatment of less serious diseases is common in this area, but other methods are less common probably due to lack of familiarity and lack of access. Further studies are suggested to investigate the causes of these differences more closely.

## Supplementary Information

**Additional file 1: Appendix 1.** Logistic Regression for Self-help practices used in the last year. **Appendix 2.** Logistic Regression for Use of complementary medicine throughout the life.

## Data Availability

The datasets used and/or analyzed during the current study are available from the corresponding author on reasonable request.

## Abbreviations

CAM: Complementary and Alternative Medicine
I-CAM-Q: International CAM Questionnaire
PM: Persian Medicine
DHS: Demographic and Health Surveys
OR: Odds Ratios
