# Supplementary material for: The prevalence of complementary and alternative medicine use in the general population of Babol, North of Iran, 2018
Source: BMC Complement Med Ther. 2021 Apr 8;21:113. doi: 10.1186/s12906-021-03281-7 (PMC8033722; doi:10.1186/s12906-021-03281-7)
Supplement: Supplementary file 1 — Additional file 1: Appendix 1. Logistic Regression for Self-help practices used in the last year. Appendix 2. Logistic Regression for Use of complementary medicine throughout the life. [file 12906_2021_3281_MOESM1_ESM.docx]

Appendix 1: **Logistic Regression for Self-help practices used in the last year**

| **Type of Self-help practices ^a^** | **Covariates** | **P-Value** | **AOR^f^** | **95% CI. for AOR** | |
| --- | --- | --- | --- | --- | --- |
|  |  |  |  | **Lower** | **Upper** |
| Cupping | Marital status ^b^ | **0.013** |  |  |  |
|  | Married | 0.544 | 1.296 | .560 | 2.999 |
|  | Divorced | 0.999 | .000 | .000 | . |
|  | Widow and widower | **0.002** | 7.293 | 2.108 | 25.232 |
| Massage | Education ^c^ | 0.508 |  |  |  |
|  | Middle School | 0.958 | 1.017 | 0.539 | 1.921 |
|  | High school | 0.401 | 1.260 | 0.734 | 2.163 |
|  | Associate Degree or bachelor's degree | 0.488 | 0.764 | 0.357 | 1.635 |
|  | Master's degree and higher | 0.998 | 0.000 | 0.000 | . |
|  | Under 7 age old | 0.127 | 0.210 | 0.028 | 1.559 |
| Diet | sex(male) ^d^ | **0.003** | 0.197 | 0.067 | 0.579 |
| Music therapy | Education ^c^ | **<0.001** |  |  |  |
|  | Middle School | **<0.001** | 4.222 | 2.385 | 7.472 |
|  | High school | **<0.001** | 3.802 | 2.198 | 6.576 |
|  | Associate Degree or bachelor's degree | **<0.001** | 4.860 | 2.694 | 8.767 |
|  | Master's degree and higher | **0.025** | 3.675 | 1.178 | 11.471 |
|  | Under 7 age old | 0.996 | .000 | .000 | . |
|  | age | **<0.001** | 0.980 | 0.968 | 0.991 |
|  | sex(male) ^d^ | **0.031** | 0.678 | 0.477 | 0.965 |
| Water therapy | Education ^c^ | **0.389** |  |  |  |
|  | Middle School | 0.674 | 1.154 | 0.593 | 2.245 |
|  | High school | 0.150 | 1.514 | 0.860 | 2.665 |
|  | Associate Degree or bachelor's degree | 0.426 | 0.723 | 0.325 | 1.606 |
|  | Master's degree and higher | 0.426 | 0.436 | 0.057 | 3.355 |
|  | Under 7 age old | 0.996 | .000 | .000 | . |
|  | location (urban) ^e^ | **0.002** | 2.142 | 1.313 | 3.494 |
|  | sex(male) ^d^ | 0.089 | 1.498 | 0.940 | 2.386 |
| Praying | age | **0.006** | 1.028 | 1.008 | 1.048 |
| Vow, charity and participating in Religious ceremonies | location (urban) ^e^ | **0.002** | 1.370 | 1.127 | 1.666 |
|  | sex(male) ^d^ | **0.028** | 0.802 | 0.659 | 0.977 |
|  | Marital status ^b^ | **<0.001** |  |  |  |
|  | Married | **<0.001** | 2.369 | 1.917 | 2.927 |
|  | Divorced | **0.006** | 5.293 | 1.629 | 17.195 |
|  | Widow and widower | **0.001** | 2.871 | 1.539 | 5.355 |

a: As response variable in multivariable logistic regression

b: The reference category is Single person

c: The reference category is illiterate or elementary school

d: The reference category is female

e: The reference category is rural location

f: Adjusted Odds Ratio

Significant p-values were showed as bold text

Appendix 2: **Logistic Regression for Use of complementary medicine throughout the life**

| **Type of Self-help practices ^a^** | **Covariates** | **P-Value** | **AOR^f^** | 95% C.I. for AOR | |
| --- | --- | --- | --- | --- | --- |
|  |  |  |  | Lower | Upper |
| Cupping | age | **<0.001** | 1.030 | 1.014 | 1.047 |
|  | sex(male)^b^ | 0.063 | 0.595 | 0.345 | 1.028 |
|  | Education ^c^ | **0.030** |  |  |  |
|  | Middle School | 0.296 | 1.629 | 0.652 | 4.073 |
|  | High school | **0.001** | 3.460 | 1.688 | 7.089 |
|  | Associate Degree or bachelor's degree | 0.057 | 2.383 | 0.973 | 5.836 |
|  | Master's degree and higher | 0.614 | 1.712 | .213 | 13.788 |
|  | Under 7 age old | 0.997 | .000 | .000 | . |
| Wet cupping | sex(male)^b^ | **<0.001** | 2.380 | 1.556 | 3.641 |
|  | Marital status ^d^ | **0.023** |  |  |  |
|  | Married | **0.011** | 1.855 | 1.153 | 2.985 |
|  | Divorced | 0.482 | 2.135 | 0.257 | 17.723 |
|  | Widow and widower | **0.013** | 4.364 | 1.357 | 14.027 |
|  | Education ^c^ | **0.002** |  |  |  |
|  | Middle School | 0.124 | 1.656 | 0.871 | 3.150 |
|  | High school | **<0.001** | 2.632 | 1.529 | 4.531 |
|  | Associate Degree or bachelor's degree | **<0.001** | 3.269 | 1.812 | 5.899 |
|  | Master's degree and higher | 0.353 | 1.821 | 0.514 | 6.445 |
|  | Under 7 age old | 0.997 | .000 | .000 | . |
| Massage | Marital status ^d^ | **0.012** |  |  |  |
|  | Married | **0.002** | 3.083 | 1.510 | 6.296 |
|  | Divorced | 0.192 | 4.162 | 0.488 | 35.498 |
|  | Widow and widower | **0.019** | 5.160 | 1.310 | 20.332 |
|  | Education ^c^ | 0.384 |  |  |  |
|  | Middle School | 0.053 | 1.993 | 0.991 | 4.008 |
|  | High school | 0.086 | 1.776 | 0.922 | 3.421 |
|  | Associate Degree or bachelor's degree | 0.060 | 2.035 | 0.969 | 4.274 |
|  | Master's degree and higher | 0.998 | .000 | .000 | . |
|  | Under 7 age old | 0.997 | .000 | .000 | . |
| Persian medicine | location (urban)^e^ | 0.068 | 0.766 | 0.574 | 1.020 |
|  | sex(male)^b^ | **0.031** | 0.735 | 0.556 | 0.971 |
|  | Education ^c^ | 0.082 |  |  |  |
|  | Middle School | **0.040** | 1.510 | 1.018 | 2.240 |
|  | High school | 0.620 | 1.102 | .750 | 1.620 |
|  | Associate Degree or bachelor's degree | **0.011** | 1.749 | 1.137 | 2.689 |
|  | Master's degree and higher | 0.149 | 1.890 | 0.797 | 4.484 |
|  | Under 7 age old | 0.321 | 1.370 | 0.736 | 2.549 |
| Music therapy | location (urban)^e^ | **0.006** | 1.812 | 1.190 | 2.758 |
|  | age | **0.030** | 0.986 | 0.973 | 0.999 |
|  | Education ^c^ | **<0.001** |  |  |  |
|  | Middle School | **<0.001** | 5.750 | 2.928 | 11.291 |
|  | High school | **<0.001** | 4.180 | 2.148 | 8.135 |
|  | Associate Degree or bachelor's degree | **0.001** | 3.642 | 1.734 | 7.649 |
|  | Master's degree and higher | 0.078 | 3.271 | 0.877 | 12.199 |
|  | Under 7 age old | 0.997 | .000 | .000 | . |
| Water therapy | location (urban)^e^ | **0.001** | 1.807 | 1.277 | 2.557 |
|  | Marital status ^d^ | **0.009** |  |  |  |
|  | Married | **0.001** | 2.017 | 1.344 | 3.028 |
|  | Divorced | 0.863 | 1.200 | 0.151 | 9.549 |
|  | Widow and widower | 0.380 | 1.638 | 0.544 | 4.928 |
|  | Education ^c^ | 0.385 |  |  |  |
|  | Middle School | 0.647 | 1.119 | 0.692 | 1.808 |
|  | High school | 0.138 | 1.369 | 0.904 | 2.075 |
|  | Associate Degree or bachelor's degree | 0.324 | 0.751 | 0.426 | 1.326 |
|  | Master's degree and higher | 0.660 | 1.251 | 0.461 | 3.396 |
|  | Under 7 age old | 0.996 | .000 | .000 | . |
| Acupuncture | Age | **0.006** | 1.031 | 1.009 | 1.054 |
|  | sex(male)^b^ | **0.019** | 0.264 | 0.087 | 0.800 |

a: As response variable in multivariable logistic regression

b: The reference category is female

c: The reference category is illiterate or elementary school

d: The reference category is Single person

e: The reference category is rural location

f: Adjusted OR

Significant p-values were showed as bold text
